# Economic stress and condescending treatment in childhood and adult self-rated health: results from a population study in Sweden

**DOI:** 10.1186/s12889-017-4438-x

**Published:** 2017-05-22

**Authors:** Fredrik Granström, Hans-Georg Eriksson, Anu Molarius

**Affiliations:** 1 Centre for Clinical Research Sörmland, Uppsala University, Eskilstuna, Sweden; 2 Division of Community Medicine, Department of Medical and Health Sciences, Linköping University, Linköping, Sweden; 3 Competence Centre for Health, Region Västmanland, 721 89 Västerås, Sweden; 4 Department of Public Health, Karlstad University, Karlstad, Sweden

**Keywords:** Childhood adversities, Life course, Self-rated health, Population studies, Sweden

## Abstract

**Background:**

Even today, 12% of the children in Sweden live in poverty and many children are exposed to adverse experiences, such as being bullied, which may have long-term consequences on public health. This study examined the associations between economic stress and condescending treatment in childhood and self-rated health (SRH) in adulthood.

**Methods:**

The study is based on 26,706 persons who responded to a postal survey questionnaire sent to a random sample of men and women aged 25–84 years in 2012 (response rate 53%). The associations between childhood circumstances and adult SRH were analysed by logistic regression, adjusting for sex, age, economic stress in adulthood, condescending treatment in adulthood, socioeconomic status and several other known material, behavioural and psychosocial risk factors.

**Results:**

In total, 39% of both men and women reported economic stress in their family during childhood. 36% of the men and 41% of the women indicated that they had been treated in a condescending manner, e.g. in school or at home, during childhood. Both economic stress in childhood and condescending treatment in childhood were strongly associated with adult SRH. The associations attenuated, but were still statistically significant after adjustment for adulthood circumstances and other risk factors.

**Conclusion:**

Economic stress in childhood and condescending treatment in childhood were associated with SRH in adulthood, both independently and through adulthood circumstances. The results underline the importance of taking into account both material and psychosocial circumstances over the whole life course when developing public health measures.

**Electronic supplementary material:**

The online version of this article (doi:10.1186/s12889-017-4438-x) contains supplementary material, which is available to authorized users.

## Background

Over the last few decades, the life course perspective, including the association between childhood circumstances and adult health, has been increasingly acknowledged as an important field of study [1, 2]. It has been suggested that social conditions accumulate from birth and onwards to build a base for the development of health during the remaining life span [3]. Thus, a child’s social conditions have both immediate health effects and long-term health consequences [4]. The latter is explained by the fact that body organs and body systems are still under development, and therefore more susceptible to adaptation to the social conditions [5]. Furthermore, the long-term effects of childhood circumstances are amplified by four separate mechanisms operating in parallel: impact on physical and emotional health; the shaping of future lifestyle; impact on the cognitive development (and thus school results); and the forming of the social identity [1]. These mechanisms are operating through complex interactions between psychological, psychosocial, social and biological factors [3]. For example, psychological stress during childhood can cause permanent problems with controlling emotions, to connect with other people and with self-esteem. Childhood social environment is also associated with health behaviour, such as smoking and heavy alcohol use, in adulthood [6, 7].

Material conditions in the family during childhood have been shown to influence adult health status in several ways. Adverse material conditions increase the risk of impaired mental well-being among the parents which, as a consequence, leads to poorer parental support for the child [8]. Furthermore, material deprivation can be related to poor diet or overcrowding, affecting sleep quality and homework environment [9]. In addition, parental socioeconomic status has been shown to be independently related to health behaviour in adulthood [10].

Previous studies have shown that economic hardship during childhood has an impact on health in adulthood, regardless of current economic conditions [11]. This has been reported for several health measures such as self-rated health (SRH) [12, 13] and mental disorders [14–16]. Similarly, associations between current economic difficulties and poor SRH [12, 13, 17] as well as impaired mental health [14, 18] have been found.

Experiences of being condescendingly treated have been shown to be strongly associated with poor SRH and mental disorders [17, 19]. The same is true for women and men who have been bullied in the workplace [20]. Condescending treatment can be seen as interpersonal discrimination which, according to social epidemiology, is a strong risk factor for poor health [21, 22]. To our knowledge there are no studies on condescending treatment during childhood and health in adulthood. However, related concepts such as childhood adversities have been investigated. For example, previous studies have shown that exposure to bullying at school is associated with poor adult SRH [23] and poor mental health in adulthood [15, 24, 25]. It also increases the risk of being exposed to bullying in the workplace as an adult, which in turn has adverse effects on health [26]. In addition, stressful relations with parents in childhood have been shown to be related to ill health among middle-aged men [27] and serious conflicts within the childhood family have been shown to be associated with poor SRH in young adulthood in women [23].

In 2013, 12% of the children in Sweden lived in poverty, defined as living in families receiving social benefit or being below a certain level of disposable income (e.g. about 1800 EUR/month for families with two adults and two children) [28]. In addition, many children are exposed to adverse experiences, for instance being bullied [29]. It is therefore important to study the associations between childhood material and psychosocial circumstances and adult health. We are not aware of previous studies that have simultaneously investigated childhood material and psychosocial factors and adult health in Sweden. According to life course epidemiology, material and psychosocial adversities in childhood increase the risk of corresponding adversities in adulthood [9]. Furthermore, adult lifestyle and social identity are shown to partly originate from childhood conditions [1, 6, 7, 10]. Adult socioeconomic status such as educational level or employment status are other possible mediating factors [23]. Thus, taking into account these possible mediating and confounding factors is necessary to elucidate the association between childhood circumstances and adult health.

The aim of this study was to examine the associations between economic stress and condescending treatment in childhood and SRH in adulthood, both independently and through adulthood circumstances, in the general adult population in Sweden.

## Methods

This study is based on a postal questionnaire (the Swedish National Public Health Survey) sent to a random population sample in Sweden in 2012. In four counties in the central part of Sweden, covering 39 municipalities with about one million inhabitants, extended population samples were drawn, stratified by gender, age group and municipality. In these counties the questionnaire included several additional questions, including questions on childhood circumstances (see Additional file 1). The sampling frame was the population register at Statistics Sweden, the statistical administrative authority in Sweden, covering all inhabitants of the study area. The survey was coordinated by the National Institute of Public Health, now The Public Health Agency of Sweden. Data collection was discontinued after two postal reminders. A total of 26,706 men and women aged 25–84 years were included in the study. The overall response rate was 53%.

### Outcome

SRH was assessed by the question “How do you rate your general health?” with the options “very good”, “good”, “fair”, “poor” and “very poor”. In the analyses, the categories “very good” and “good” were combined as well as the categories “poor” and “very poor” to avoid categories with small number of subjects.

### Childhood circumstances

Economic stress in childhood was measured by the question “Did your family experience any economic difficulties when you grew up?” with the answer options “no”, “yes, during minor part of childhood”, “yes, during major part of childhood” and “yes, during the entire childhood”. Condescending treatment in childhood was assessed by the question “Were you treated in a condescending manner while you were growing up, for example in school or at home?”. The answer categories were “no”, “yes, sometimes” and “yes, often”.

### Adulthood circumstances

Economic stress in adulthood was measured by a survey question on difficulty with covering expenses for food, rent, bills etc. during the past 12 months, with the answer options “no”, “yes, once”, “yes, several times”. Condescending treatment in adulthood was assessed by the question “Have you during the past three months at any time experienced that you have been treated in a condescending manner by anyone?”. The answer categories were “no”, “yes, occasionally”, and “yes, several times”.

### Sociodemographic factors

Information on sex, age, educational level and country of birth were obtained from official registers. *Educational level* was categorized into three levels: low (elementary school), medium (upper secondary school), and high (at least 3 years of university or corresponding education). *Country of birth* was categorized into born in Sweden, born in another Nordic country, born in another European country and born outside of Europe. *Employment status* was derived from a survey question about whether the respondent was employed, self-employed, a student, unemployed, on sickness leave, on disability pension or retired.

### Psychosocial factors

S*ocial support* was assessed by the question: “Can you get help from any person or persons if you have practical problems or are ill? E.g. get advice, borrow things, help with shopping, repairs etc.”. The response options were: “Yes, always”, “Yes, mostly”, “No, mostly not” and “No, never”. *Burdensome domestic work* was measured by a question on how often the respondent experienced domestic work as a burden (“all or most of the time”, “sometimes”, “seldom”, “never”).

### Behavioural factors

The level of *physical activity* was measured with the question: "How much physical movement and exertion have you had in the last 12 months?", with the options: “Sedentary leisure time (<2h a week)”, “Moderate exercise in leisure time (⩾2h a week)”, “Moderate, regular exercise in leisure time (sweat-inducing exercise at least for 30 minutes at a time 1–2 times a week)” and “Regular exercise and training (at least three times a week)”. *Daily smoking* was measured by the question “Do you smoke daily?” (yes/no). *Risk consumption of alcohol* was measured by an index of three question (AUDIT-C) developed to identify “persons with hazardous and harmful patterns of alcohol consumption” [30]. The cut-offs for risk consumption in this study were 6 points or more for women and 8 points or more for men [31].

The individuals in the survey were informed that completed questionnaires would be linked to the Swedish official registries through the personal identification numbers, in order to access information on sex, age, geographic area, educational level and country of birth. Thus, the respondents gave their informed consent to the linking of registry data. Immediately after record linkage, the personal identification numbers were deleted. Statistics Sweden carried out the sampling and data collection and linkage with registry data, and delivered the de-identified data to the county councils. The survey was approved by the Regional Board of Ethics, Uppsala (EPN 2012/256).

#### Statistical analyses

Crude proportions reporting childhood economic stress and condescending treatment in childhood were calculated by sex, age, educational level, country of birth, employment status, current economic stress, current condescending treatment and other covariates. Differences in proportions were tested with chi-squared statistics. Also, distributions of SRH were calculated by sex, age, childhood economic stress and condescending treatment in childhood. To take into account circumstances in adulthood and other risk factors for SRH, multivariate analyses were carried out using multinomial logistic regression. The results are reported as odds ratios (OR) and 95% confidence intervals (95% CI) for having poor and fair SRH, compared to good SRH. Since there was a moderate correlation (Spearman’s rho = 0.31) between childhood economic stress and condescending treatment in childhood these variables were added simultaneously in all models. In the first model, ORs for childhood economic stress and condescending treatment in childhood were adjusted for sex and age. In the second model, the ORs were adjusted for economic stress and condescending treatment in adulthood. In the third model, further adjustment was made for educational level, employment status, country of birth, social support, burdensome domestic work, smoking, high-risk alcohol consumption, and physical activity.

## Results

In total, 39% of both men and women reported economic stress in their family at least during some part of their childhood (Table 1). Similar proportions, 36% of the men and 41% of the women, indicated that they had been treated in a condescending manner sometimes or often, e.g. in school or at home, during childhood. Economic stress during childhood was more common among persons with low educational level, those born outside Sweden, the unemployed and those on disability pension. Condescending treatment in childhood was most common among the unemployed and those on disability pension. Differences in the prevalence of childhood conditions were also found by adulthood circumstances and the behavioural and psychosocial risk factors included in the analyses.

**Table 1:** Crude distribution (%) of economic stress in childhood and condescending treatment in childhood by sex, educational level, country of birth, employment, current condescending treatment, current economic stress, social support, burdensome domestic work, daily smoking, risk consumption of alcohol and physical activity, 25–84 years

		Economic stress in childhood					Condescending treatment in childhood			
	N	Entire childhood	Major part	Minor part	No	*p*-value*	Yes, often	Yes, sometimes	No	*p*-value*
Total	26,706	6.8	11.8	20.0	61.4		7.7	31.1	61.2	
Sex						<.001				<.001
Men	12,221	6.2	11.5	21.1	61.3		6.1	30.4	63.5	
Women	14,485	7.4	12.0	19.1	61.4		9.1	31.6	59.3	
Educational level						<.001				<.001
Compulsory	5617	9.8	14.7	19.6	55.9		7.2	27.2	65.6	
Secondary	11,863	6.9	11.9	20.2	61.0		8.4	31.7	60.0	
Post-secondary	9097	4.8	9.9	19.9	65.4		7.1	32.7	60.2	
Country of birth						<.001				<.001
Sweden	23,078	5.6	10.9	19.1	64.4		7.5	30.9	61.7	
Other Nordic country	1593	14.5	18.0	23.7	43.9		10.5	33.1	56.4	
Outside Nordic countries	2035	14.4	17.6	27.1	40.9		8.3	32.0	59.7	
Employment						<.001				<.001
Employed	12,271	5.0	10.5	20.2	64.3		8.0	34.1	58.0	
Self-employed	1668	5.0	10.6	18.7	65.7		6.8	30.9	62.3	
Student	500	9.4	12.6	24.6	53.4		14.1	37.2	48.7	
Unemployed	722	11.9	13.0	22.6	52.5		17.5	36.6	45.9	
On sickness leave	621	7.6	14.2	21.3	57.0		13.8	32.5	53.6	
On disability pension	813	14.3	16.4	20.5	48.8		23.2	34.1	42.7	
Retired	8667	7.9	12.7	19.8	59.6		4.4	25.7	69.8	
Condescently treated during the last 3 months						<.001				<.001
Yes, several times	883	15.2	16.5	20.7	47.6		34.7	36.5	28.8	
Yes, occasionally	6246	8.6	14.3	22.2	54.9		14.0	43.9	42.1	
No	19,497	5.9	10.8	19.2	64.1		4.4	26.7	68.8	
Economic stress past 12 months						<.001				<.001
Yes, several times	1888	16.8	20.4	22.1	40.7		23.1	38.6	38.4	
Yes, once	1317	10.7	14.6	24.4	50.3		12.8	38.0	49.2	
No	23,330	5.8	10.9	19.6	63.7		6.2	30.1	63.8	
Social support						<.001				<.001
No, never	459	18.5	13.3	20.3	47.9		20.8	31.3	47.9	
No, mostly not	912	14.1	19.5	24.1	42.2		18.4	38.8	42.8	
Yes, mostly	7095	8.1	15.0	23.1	53.7		9.9	36.9	53.2	
Yes, always	18,004	5.6	10.2	18.6	65.6		6.0	28.5	65.5	
Burdensome domestic work						<.001				<.001
All or most of the time	1857	14.5	16.3	19.1	50.1		21.6	34.1	44.3	
Never/seldom/sometimes	24,508	6.2	11.5	20.1	62.2		6.7	30.9	62.4	
Daily smoker						<.001				<.001
Yes	3146	9.8	14.8	21.2	54.2		11.8	31.5	56.6	
No	23,346	6.4	11.4	19.8	62.4		7.2	31.0	61.8	
Risk consumption of alcohol						.002				<.001
Yes	1166	8.2	14.2	21.0	56.6		13.0	34.6	52.5	
No	24,465	6.7	11.6	20.0	61.7		7.5	31.0	61.5	
Physical activity						<.001				<.001
Sedentary	3335	11.0	14.5	19.2	55.3		10.7	30.8	58.5	
Moderate	12,655	6.5	11.9	20.2	61.5		7.2	30.6	62.2	
Moderate regular	5906	5.8	11.2	20.4	62.7		6.9	32.0	61.1	
Regular exercise	4438	5.8	10.2	19.3	64.7		7.6	31.7	60.7	

*p-value based on chi-squared test

**Fig. 1:**
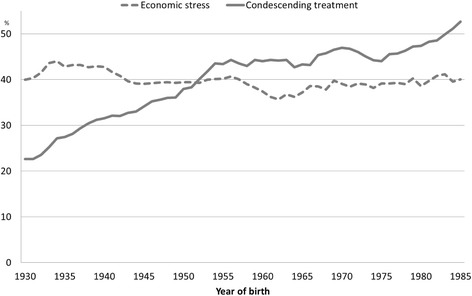
Proportions reporting economic stress in childhood and condescending treatment in childhood by year of birth

When different age groups were compared, the proportions reporting economic stress during childhood were similar (Fig. 1). However, the proportion that reported that they were condescendingly treated in childhood was largest in the youngest age groups and smallest among the elderly.

In total, 68% of the respondents reported good SRH. The proportions with good SRH decreased by the frequency of exposure to economic stress and condescending treatment in childhood (Table 2). Only 50% of the respondents who reported economic stress during the entire childhood had good SRH in adulthood. Similarly, only 51% of those often exposed to condescending treatment in childhood had good SRH.

**Table 2:** Crude distribution (%) of self-rated health by sex, age, economic stress in childhood and condescending treatment in childhood

	N	Poor	Fair	Good	*p*-value*
Total	25,951	6.2	26.2	67.6	
Sex					<.001
Men	11,893	5.4	25.8	68.8	
Women	14,058	6.9	26.5	66.6	
Age					<.001
25–34	2555	3.7	14.0	82.3	
35–49	5700	5.6	18.8	75.6	
50–64	7499	7.4	24.9	67.7	
65–84	10,197	6.4	34.4	59.4	
Economic stress in childhood svårigheter under uppväxten					<.001
Yes, during the entire childhood	1756	14.8	35.6	49.5	
Yes, during major part	3069	9.2	32.9	57.8	
Yes, during minor part	5191	5.9	27.9	66.2	
No	15,935	4.8	23.2	71.9	
Condescently treated in childhood					<.001
Yes, often	1980	16.7	32.2	51.1	
Yes, sometimes	8067	6.5	28.9	64.6	
No	15,813	4.8	23.9	71.3	

*p-value based on chi-squared test.

The results of the sex- and age-adjusted logistic regression analyses show that both economic stress in childhood and condescending treatment in childhood were strongly associated with poor SRH in adulthood (Table 3). Respondents reporting economic stress during the entire childhood (OR = 2.30, 95% CI:2.29–3.25) or the major part (OR = 1.83, 95% CI:1.57–2.13) had increased risk of poor SRH in adulthood. Similarly, respondents reporting that they were exposed to condescending treatment during childhood had increased risk, especially those who reported that they often were condescendingly treated (OR = 4.20, 95% CI:3.57–4.94). The associations were considerably attenuated, but still statistically significant [OR = 2.17 (95% CI:1.81–2.59) for economic stress during the entire childhood, OR = 1.59 (95% CI:1.36–1.87) for major part of the childhood and OR = 2.60 (95% CI:2.18–3.10) for having often been condescendingly treated in childhood], when adjusted for adulthood circumstances. Current economic stress and condescending treatment had stronger associations with poor SRH than childhood conditions. After adjustment for socioeconomic status, psychosocial and behavioural risk factors, the associations between the childhood circumstances and adult SRH were further attenuated, but remained statistically significant [OR = 1.41 (95% CI:1.14–1.76) for economic stress during the entire childhood, OR = 1.33 (95% CI:1.11–1.60) for major part of the childhood and OR = 1.99 (95% CI:1.61–2.45) for having often been condescendingly treated in childhood].

**Table 3:** Odds ratios for poor and fair self-rated health in relation to condescending treatment in childhood and economic stress in childhood, 25–84 years

		Model 1	Model 2	Model 3
Self-rated health (reference category = good)		OR (95% CI)	OR (95% CI)	OR (95% CI)
Poor	Economic stress in childhood svårigheter under uppväxten			
(*n* = 1610)	Yes, during the entire childhood	2.73 (2.29–3.25)	2.17 (1.81–2.59)	1.41 (1.14–1.76)
	Yes, during major part of the childhood	1.83 (1.57–2.13)	1.59 (1.36–1.87)	1.33 (1.11–1.60)
	Yes, during minor part of the childhood	1.20 (1.04–1.38)	1.11 (0.96–1.28)	1.02 (0.86–1.20)
	No	Ref	Ref	Ref
	Condescently treated in childhood			
	Yes, often	4.20 (3.57–4.94)	2.60 (2.18–3.10)	1.99 (1.61–2.45)
	Yes, sometimes	1.43 (1.27–1.62)	1.23 (1.08–1.39)	1.27 (1.10–1.47)
	No	Ref	Ref	Ref
	Economic stress past 12 months			
	Yes, several times		5.54 (4.74–6.48)	2.57 (2.11–3.13)
	Yes, once		3.07 (2.51–3.76)	2.01 (1.57–2.57)
	No		Ref	Ref
	Condescently treated during the last 3 months			
	Yes, several times		4.40 (3.54–5.47)	3.27 (2.50–4.28)
	Yes, occasionally		1.81 (1.59–2.06)	1.92 (1.65–2.24)
	No		Ref	Ref
Fair	Economic stress in childhood svårigheter under uppväxten			
(*n* = 6753)	Yes, during the entire childhood	1.71 (1.52–1.92)	1.55 (1.38–1.75)	1.33 (1.16–1.52)
	Yes, during major part of the childhood	1.50 (1.37–1.64)	1.40 (1.28–1.54)	1.29 (1.16–1.42)
	Yes, during minor part of the childhood	1.23 (1.14–1.33)	1.18 (1.09–1.27)	1.13 (1.04–1.22)
	No	Ref	Ref	Ref
	Condescently treated in childhood			
	Yes, often	2.09 (1.86–2.34)	1.65 (1.46–1.86)	1.41 (1.24–1.61)
	Yes, sometimes	1.39 (1.31–1.49)	1.28 (1.19–1.36)	1.28 (1.19–1.38)
	No	Ref	Ref	Ref
	Economic stress past 12 months			
	Yes, several times		2.43 (2.16–2.73)	1.64 (1.44–1.88)
	Yes, once		1.74 (1.52–1.99)	1.49 (1.29–1.74)
	No		Ref	Ref
	Condescently treated during the last 3 months			
	Yes, several times		1.98 (1.67–2.36)	1.76 (1.46–2.13)
	Yes, occasionally		1.53 (1.42–1.65)	1.58 (1.45–1.71)
	No		Ref	Ref

Model 1: Adjusted for sex and age (*n* = 25,860)

Model 2: Adjusted for sex, age, economic stress in adulthood and condescending treatment in adulthood (*n* = 25,662)

Model 3: Adjusted for sex, age, economic stress in adulthood, condescending treatment in adulthood, employment status, educational level, country of birth, social support, burdensome domestic work, daily smoking, risk consumption of alcohol and physical activity (*n* = 23,869)

## Discussion

The results of this study indicate that both economic stress in childhood and condescending treatment in childhood are associated with SRH in adulthood. The associations were attenuated, but still statistically significant, when adjusted for economic stress and condescending treatment in adulthood and other risk factors for poor SRH. Those who reported economic stress during the entire childhood or during major part of the childhood had an increased risk for poor SRH. In addition, those who were most often exposed to condescending treatment had the highest risk of poor SRH.

The associations between current economic stress or recent exposure to condescending treatment and adult SRH were stronger than the associations between childhood circumstances and adult SRH. Previous studies have shown that current economic difficulties [12, 32, 33] and recent condescending treatment [17] are strongly associated with poor SRH among adults. Also, the result that childhood economic stress was independently associated with poor SRH is in line with results from previous studies [11, 13]. We are not aware of previous studies examining the health impacts of being condescendingly treated in childhood, but studies have shown that severe emotional stress in childhood, which might be a result of condescending treatment, can permanently increase the susceptibility to ill health [34, 35]. Previous studies have also shown that exposure to bullying at school is associated with poor health in adulthood [15, 23–25].

Theories aiming at explaining how early life conditions affect health suggest that the impact is amplified by several parallel mechanisms [1], and that these are operating in complex interaction processes [3]. In the current study, we investigated how two different types of childhood circumstances independently contribute to explain adult health status. Since there was a moderate correlation between economic stress and condescending treatment in childhood these factors were adjusted for each other in all logistic regression models. We also showed that some of the contributions were mediated by adult circumstances and other risk factors, e.g. related to health behaviour [1]. These findings emphasize the importance of the life course perspective, defined as “the long term effects on later health or disease risk of physical or social exposures during gestation, childhood, adolescence, young adulthood and later adult life” [5]. In addition, childhood circumstances have been shown to contribute substantially to health inequalities between people of different educational levels [36]. In young adulthood, a large part of this contribution seems to be explained by behavioural factors and only a minor part by current circumstances [36].

Socioeconomic factors were associated with economic stress and condescending treatment during childhood. Childhood economic stress was more common among persons with low educational level, those born outside Sweden, the unemployed and those on disability pension. Similar results for country of birth and employment have been reported from the southern part of Sweden (Skåne) [13]. Condescending treatment in childhood was most common among the unemployed and those on disability pension. In the final model, where the results were adjusted for socioeconomic factors, the odds ratios were attenuated, indicating that part of the associations between childhood conditions, in particular economic stress, and adult SRH are mediated by adult socioeconomic status such as educational level and employment.

There were small differences in the prevalence of childhood economic stress by age, even though the prosperity level of the country differed remarkably between the interwar period, when the oldest respondents grew up, and the second half of the twentieth century. This may reflect the fact that people interpret economic stress in relative terms, i.e. measured by their income rank in comparison to others rather than being below any specific absolute level of income [37]. A lower response rate in the younger age groups may also have played a role. However, reporting of condescending treatment in childhood differed between age groups. The younger age groups reported higher levels of condescending treatment in childhood. This may be due to real differences in exposure between age groups, due to different interpretations of the term “condescending treatment” in different generations or maybe an indication that memories of condescending treatment fade with time. However, a higher prevalence of bullying at school among younger adults (18–29 years) than among those 30–39 years of age has also been reported from a representative population study in Finland [23].

Both economic stress in childhood and condescending treatment, e.g. in school or at home, during childhood were relatively common in this adult general population, about four in ten reported these childhood circumstances. As both these factors were independently associated with adult SRH, public health measures addressed to both material and psychosocial circumstances in childhood are needed to promote the future health in the population. The prevalence of self-reported economic stress during the major part of the childhood, shown to be a particular risk factor for poor SRH in adulthood, was about 17%. This is somewhat higher than the current Swedish childhood poverty rate of 12%, based on register information [28]. Nevertheless, our findings are in line with the notion that childhood poverty of today may have long-lasting negative consequences for health in the general population.

### Strengths and limitations

Conducting retrospective measurements by asking the respondent to report factors in childhood can lead to increased uncertainty compared to longitudinal studies, where all data are collected at the time of exposure. We presume, however, that both childhood economic stress and childhood condescending treatment are such significant experiences that the memories of those are likely to prevail into adulthood, especially if the experiences influenced a large part of the childhood. Moreover, also most previous studies in this field have been constrained to use retrospective data, as objective, prospective data on childhood circumstances are scarce. An additional risk by asking about circumstances in the past is that memories can be influenced by prevailing conditions at the time of reporting, i.e. the so-called recall bias. For instance, if the respondent is experiencing hard current life conditions, it is more likely that memories of difficulties in childhood come into mind [38]. However, in the present study almost half of those, who reported condescending treatment in childhood, reported no condescending treatment during the past 3 months, suggesting that recall bias was not a major problem. In addition, a previous evaluation of the method of examining childhood economic stress retrospectively reported good validity [39].

A further limitation of the study is that both the risk factors and the outcome were self-reported and that the validity of the risk factor measures is uncertain. However, experiences of discrimination are usually measured through self-reports and different studies have used different measures of perceived discrimination since there is a lack of validated instruments to be used in large scale epidemiologic studies [21, 22]. Furthermore, previous studies on childhood circumstances and adult health have based their results on self-reported data, with dichotomous measures or measures with a few categories, on economic stress in childhood [12, 13, 23] and for example on bullying at school [23, 24, 36].

The response rate in the study was 53%. Non-response is more common in groups that are economically disadvantaged, such as persons with low educational level and those born outside the Nordic countries [40]. It is therefore possible that the prevalence of childhood economic stress, and perhaps even condescending treatment in childhood, may be underestimated in our study. However, it is unlikely that non-response would have explained the associations found between childhood circumstances and SRH.

A strength of the current study is that we investigated two distinct types of childhood conditions, one material and one psychosocial, simultaneously. A further strength is that the study was based on a representative sample of the adult population in a large area in Sweden with a similar distribution of SRH as in the nationally representative sample in Sweden [31]. The study includes different types of municipalities and residential areas as well as different socio-economic conditions. The sample covers a wide age range, from young adults to old-age pensioners, with varying childhood conditions. An additional strength is the use of SRH as health outcome, as it is a well-known and validated measurement [41].

## Conclusion

To our knowledge, this is the first population based study in Sweden to show that both economic stress in childhood and condescending treatment in childhood are relatively common in the general population and that they are associated with SRH in adulthood, even when adjusted for current circumstances and other risk factors. The results underline the importance of taking into account both *material and psychosocial circumstances* over the whole life course when developing public health measures. Thus, efforts to reduce childhood poverty and to promote good psychosocial conditions in childhood should have positive long-lasting beneficial effects on public health.

## Additional file

Additional file 1: Life and health long questionnaire 2012.pdf; Survey questionnaire 2012. (PDF 140 kb)

## Abbreviation

SRH: Self-rated health
