# Supplementary material for: Economic stress and condescending treatment in childhood and adult self-rated health: results from a population study in Sweden
Source: BMC Public Health. 2017 May 22;17:489. doi: 10.1186/s12889-017-4438-x (PMC5441006; doi:10.1186/s12889-017-4438-x)
Supplement: Additional file 1: — Life and health long questionnaire 2012.pdf; Survey questionnaire 2012. (PDF 140 kb) [file 12889_2017_4438_MOESM1_ESM.pdf]

Health

1. How would you assess your general state of health?

1 ☐ Very good
2 ☐ Good
3 ☐ Fair
4 ☐ Poor
5 ☐ Very poor

2. If you think about your <u>physical health</u>, how many days *in the last 30 day period* would you say that it was not good (because of illness, bodily discomfort or injury)?
Number of days between 0 and 30

☐☐ **days**

3. If you think about your <u>mental health</u>, how many days *in the last 30 day period* would you say that it was not good (because of stress, depression or unease, for example)?
Number of days between 0 and 30

☐☐ **days**

4. How many days in the last 30 day period has poor <u>physical or mental health</u> hindered your ability to work or your day-to-day activities?
Number of days between 0 and 30

☐☐ **days**

5. Have you had any accidents in the last three months that led to your seeking health care or dental care?

1 ☐ No
2 ☐ Yes, once
3 ☐ Yes, more than once

6. Do you have any long-term illness, discomfort following an accident, any reduced physical function or any other long-term health problem?

1 ☐ No
2 ☐ Yes → **Does this condition mean that your ability to work is reduced *or* hinders you in your other day-to-day jobs?**

1 ☐ No, not at all
2 ☐ Yes, to some extent
3 ☐ Yes, a great deal

7. Can you see and make out normal text in daylight without difficulty?

1 ☐ Yes, without glasses
2 ☐ Yes, with glasses
3 ☐ No

8. Can you hear what is being said in a conversation between several persons without difficulty?

1 ☐ Yes, without a hearing aid
2 ☐ Yes, with a hearing aid
3 ☐ No

9. Can you run a short distance (about 100 metres)?

1 ☐ Yes → Go on to question 11
2 ☐ No

10. Are you limited in any of the following activities because of your state of health?
Mark one alternative on each row.

	Yes 1	No 2
a) Can you walk up steps without difficulty? *e.g. steps up to a bus or train*	☐	☐
b) Can you take a short walk (about 5 minutes) at a reasonably fast pace?	☐	☐
c) Do you need any aids or the help of another person to move around outdoors?	☐	☐

11. Do you have any of the following illnesses?

a) Diabetes?

1 ☐ No
2 ☐ Yes, but with no discomfort
3 ☐ Yes, minor discomfort
4 ☐ Yes, severe discomfort

b) Asthma?

1 ☐ No
2 ☐ Yes, but with no discomfort
3 ☐ Yes, minor discomfort
4 ☐ Yes, severe discomfort

c) Allergies?

1 ☐ No
2 ☐ Yes, but with no discomfort
3 ☐ Yes, minor discomfort
4 ☐ Yes, severe discomfort

d) High blood pressure?

1 ☐ No
2 ☐ Yes, but with no discomfort
3 ☐ Yes, minor discomfort
4 ☐ Yes, severe discomfort

12. How tall are you?
Answer in whole centimetres.

☐☐☐ **cm**

13. How much do you weigh?
Answer in whole kilos. If you are pregnant, state how much you normally weigh.

☐☐☐ **kg**

14. Do you have any of the following discomfort or symptoms?

a) Aches in your shoulders or neck?

1 ☐ No
2 ☐ Yes, minor discomfort
3 ☐ Yes, severe discomfort

b) Aches or pains in your back, hip pain or sciatica?

1 ☐ No
2 ☐ Yes, minor discomfort
3 ☐ Yes, severe discomfort

c) Aches or pains in your hands, elbows, legs or knees?

1 ☐ No
2 ☐ Yes, minor discomfort
3 ☐ Yes, severe discomfort

d) Headaches or migraine?

1 ☐ No
2 ☐ Yes, minor discomfort
3 ☐ Yes, severe discomfort

e) Anxiety, unease or fear?

1 ☐ No
2 ☐ Yes, minor discomfort
3 ☐ Yes, severe discomfort

f) Tiredness?

1 ☐ No
2 ☐ Yes, minor discomfort
3 ☐ Yes, severe discomfort

g) Sleeping difficulties?

1 ☐ No
2 ☐ Yes, minor discomfort
3 ☐ Yes, severe discomfort

h) Eczema or skin eruptions?

1 ☐ No
2 ☐ Yes, minor discomfort
3 ☐ Yes, severe discomfort

i) Ringing in your ears (tinnitus)?

1 ☐ No
2 ☐ Yes, minor discomfort
3 ☐ Yes, severe discomfort

j) Incontinence (urine leakage)?

1 ☐ No
2 ☐ Yes, minor discomfort
3 ☐ Yes, severe discomfort

k) Recurrent stomach or bowel problems?

1 ☐ No
2 ☐ Yes, minor discomfort
3 ☐ Yes, severe discomfort

l) Overweight, obesity?

1 ☐ No
2 ☐ Yes, minor discomfort
3 ☐ Yes, severe discomfort

Well-being

15. Have you recently been able to concentrate on whatever you're doing?

1 ☐ Better than usual
2 ☐ Same as usual
3 ☐ Less than usual
4 ☐ Much less than usual

16. Have you recently been able to enjoy your normal day-to-day activities?

1 ☐ More so than usual
2 ☐ Same as usual
3 ☐ Less so than usual
4 ☐ Much less than usual

17. Have you recently lost much sleep over worry?

1 ☐ Not at all
2 ☐ No more than usual
3 ☐ Rather more than usual
4 ☐ Much more than usual

18. Have you recently been able to face up to your problems?

1 ☐ More so than usual
2 ☐ Same as usual
3 ☐ Less able than usual
4 ☐ Much less able

19. Have you recently felt you are playing a useful part in things?

1 ☐ More so than usual
2 ☐ Same as usual
3 ☐ Less useful than usual
4 ☐ Much less useful

20. Have you recently been feeling unhappy and depressed?

1 ☐ Not at all
2 ☐ No more than usual
3 ☐ Rather more than usual
4 ☐ Much more than usual

21. Have you recently felt capable of making decisions about things?

1 ☐ More so than usual
2 ☐ Same as usual
3 ☐ Less so than usual
4 ☐ Much less capable

22. Have you recently been losing confidence in yourself?

1 ☐ Not at all
2 ☐ No more than usual
3 ☐ Rather more than usual
4 ☐ Much more than usual

23. Have you recently felt constantly under strain?

1 ☐ Not at all
2 ☐ No more than usual
3 ☐ Rather more than usual
4 ☐ Much more than usual

24. Have you recently been thinking of yourself as a worthless person?

1 ☐ Not at all
2 ☐ No more than usual
3 ☐ Rather more than usual
4 ☐ Much more than usual

25. Have you recently felt you couldn't overcome your difficulties?

1 ☐ Not at all
2 ☐ No more than usual
3 ☐ Rather more than usual
4 ☐ Much more than usual

26. Have you recently been feeling reasonably happy, all things considered?

1 ☐ More so than usual
2 ☐ About same as usual
3 ☐ Less so than usual
4 ☐ Much less than usual

27. Do you feel stressed at present?
By stressed, we mean a condition where you feel tense, restless, nervous, uneasy or unable to concentrate.

1 ☐ Not at all
2 ☐ To some extent
3 ☐ Quite a lot
4 ☐ Very much

28. Have you at any time in the last 12 months been in a situation where you have seriously considered taking your own life?

1 ☐ No
2 ☐ Yes, once
3 ☐ Yes, more than once

29. Have you at any time in the last 12 months attempted to take your own life?

1 ☐ No
2 ☐ Yes, once
3 ☐ Yes, more than once

Medicines

30. Have you, during the *last three months* taken any of the following medicines?
Mark one alternative on each row.

		No 1	Yes 2
a)	Medicine for stomach ulcer/intestinal catarrh	☐	☐
b)	Medicine for asthma or allergies	☐	☐
c)	Medicine for diabetes	☐	☐
d)	Medicine to reduce blood pressure	☐	☐
e)	Medicine for sleeplessness/insomnia	☐	☐
f)	Anti depressive medicine	☐	☐
g)	Tranquilisers/medicines to lessen anxiety	☐	☐
h)	Pain relief medicine on prescription	☐	☐
i)	Pain relief medicine without prescription	☐	☐
j)	Medicine to reduce blood lipids	☐	☐
k)	Other medicine	☐	☐

31. a) Have you, during *the last three months* refrained from buying medicine for which you had a prescription?

1 ☐ No ➡ *Go on to question 32*
2 ☐ Yes

b) What is the main reason why you did not obtain the medicine?
More than one answer can be given.

1 ☐ Became well
1 ☐ Could not afford it
1 ☐ Had sufficient medicine already
1 ☐ Too far to the pharmacy
1 ☐ Did not think the medicine would help
1 ☐ Other reason

Health care contacts

32. During the last three months, have you visited or been visited by any of the following?
Applies to your own illness or condition. Mark one alternative on each row.

		No	Yes, once	Yes, more than once
		1	2	3
a)	Doctor at the hospital	☐	☐	☐
b)	Doctor at health centre, private/company doctor or similar	☐	☐	☐
c)	District nurse	☐	☐	☐
d)	Youth clinic	☐	☐	☐
e)	Welfare officer	☐	☐	☐
f)	Psychologist	☐	☐	☐
g)	Physiotherapist	☐	☐	☐
h)	Naprapath, chiropractor, homeopath or similar	☐	☐	☐
i)	Been admitted to hospital	☐	☐	☐

33. a) Have you during the last three months believed yourself to be in need of medical care but refrained from seeking care?

1 ☐ No → Go on to question 34
2 ☐ Yes

b) What was/were the reason(s) why you did not seek medical care?
More than one answer can be given.

1 ☐ The problem cleared up
1 ☐ Waiting times too long
1 ☐ Difficult to get through on the telephone
1 ☐ Did not get an appointment quickly enough
1 ☐ Negative experience from previous visits
1 ☐ Financial reasons
1 ☐ Did not have time
1 ☐ Did not know where to go
1 ☐ Other reason

Dental health

34. How is your dental health?

1 ☐ Very good
2 ☐ Quite good
3 ☐ Neither good nor poor
4 ☐ Quite poor
5 ☐ Very poor

35. When were you last at the dentist/dental hygienist?

1 ☐ Less than a year ago
2 ☐ Between one and two years ago
3 ☐ Between three and five years ago
4 ☐ More than five years ago
5 ☐ Have never been to a dentist/dental hygienist
6 ☐ Don't know/can't remember

36. a) Have you during *the last three months* believed yourself to be in need of dental care but refrained from seeking care?

1 ☐ No → *Go on to question 37*
2 ☐ Yes

b) What was/were the reason(s) why you did not seek dental care?
More than one answer can be given.

1 ☐ The problem cleared up
1 ☐ Financial reasons
1 ☐ Declined to go (fear of dentists)
1 ☐ Did not have time
1 ☐ Other reason

Physical activity

37. How much physical movement and exertion have you had *in the last 12 months*?
If your level of activity varies, e.g. between summer and winter, try to find an average. Please mark only one alternative!

1 ☐ Sedentary leisure time

You mostly spend your free time with reading, TV, cinema or other sedentary pastimes. You walk, cycle or otherwise exercise less than 2 hours a week.

2 ☐ Moderate exercise in leisure time

You walk, cycle or otherwise exercise at least 2 hours a week, usually without sweating. Include in this walking to and from work, other walking, ordinary gardening, fishing, table tennis, bowling.

3 ☐ Moderate, regular exercise in leisure time

You exercise regularly 1-2 times a week for at least 30 minutes each time, running, swimming, tennis, badminton or other activity that makes you sweat.

4 ☐ Regular exercise and training

You exercise by e.g. running, swimming, tennis, badminton, gymnastics or similar on average at least 3 times a week. This lasts for at least 30 minutes each time.

38. How much time do you spend in a normal week in moderately strenuous activities that make you warm?
For example walking fast, gardening, heavy housework, cycling, swimming. This may vary during the year, but try to give some kind of average. Choose one alternative.

1 ☐ 5 hours or more a week
2 ☐ More than 3 hours a week and less than 5
3 ☐ Between 1 and 3 hours a week
4 ☐ No more than one hour a week
5 ☐ Not at all

39. Do you want to increase your physical activity?

1 ☐ Yes, and I believe I will be able to do this myself
2 ☐ Yes, but I need support
3 ☐ No

Food habits

40. a) How often do you eat vegetables and root vegetables?
This means all kinds of vegetables, leguminous plants and root vegetables (but not potatoes). Includes fresh, frozen, preserved, cooked, vegetable juice, vegetable soups etc. This may vary during the year, but try to give some kind of average. Choose one alternative.

1 ☐ 3 times a day or more
2 ☐ Twice a day
3 ☐ Once a day
4 ☐ 5-6 times a week
5 ☐ 3-4 times a week
6 ☐ 1-2 times a week
7 ☐ A few times a month or never

b) How often do you eat fruit and berries?
Applies to all types of fruit and berries (fresh, frozen, preserved, juice, compote etc.) This may vary during the year, but try to give some kind of average. Choose one alternative.

1 ☐ 3 times a day or more
2 ☐ Twice a day
3 ☐ Once a day
4 ☐ 5-6 times a week
5 ☐ 3-4 times a week
6 ☐ 1-2 times a week
7 ☐ A few times a month or never

41. Do you want to increase your intake of fruit and vegetables?

1 ☐ Yes, and I believe I will be able to do this myself
2 ☐ Yes, but I need support
3 ☐ No

Smoking and snuff habits

These questions apply to tobacco products such as cigarettes, cigarillos, cigars, pipe tobacco and snuff.

42. Do you smoke every day?

1 ☐ Yes *Go on to question 45*
2 ☐ No

43. Do you smoke now and then?

1 ☐ No
2 ☐ Yes

44. Have you previously smoked daily for at least 6 months?

1 ☐ No
2 ☐ Yes

45. Do you want to stop smoking?

1 ☐ I don't smoke
2 ☐ Yes, and I believe I will be able to do this myself
3 ☐ Yes, but I need support
4 ☐ No

46. Do you use snuff every day?

1 ☐ Yes *Go on to question 49*
2 ☐ No

47. Do you use snuff now and then?

1 ☐ No
2 ☐ Yes

48. Have you previously used snuff daily for at least 6 months?

1 ☐ No
2 ☐ Yes

49. Do you want to stop using snuff?

1 ☐ I don't use snuff
2 ☐ Yes, and I believe I will be able to do this myself
3 ☐ Yes, but I need support
4 ☐ No

50. How often are you indoors in places where people are smoking or have just been smoking?
Mark one alternative on each row.

	Every day<br>1	A few times a week<br>2	A few times a month<br>3	Less often or never<br>4
a) In your home	☐	☐	☐	☐
b) At work	☐	☐	☐	☐
c) In a café, bar or restaurant	☐	☐	☐	☐
d) In other enclosed places, such as in friends' homes, in the car	☐	☐	☐	☐

51. Have you ever smoked a hookah?
Mark one or several alternatives.

1 ☐ No → *Go on to question 53*
1 ☐ Yes, with nicotine
1 ☐ Yes, without nicotine
1 ☐ Yes, but don't know whether it contained nicotine

52. How often in the last 12 months have you smoked a hookah?

1 ☐ Never
2 ☐ Once
3 ☐ 2 - 6 times
3 ☐ 7 - 12 times
4 ☐ More than 12 times

53. Have you ever used hash or marijuana?

1 ☐ No
2 ☐ Yes, more than 12 months ago
3 ☐ Yes, in the last 12 months
4 ☐ Yes, in the last 30 days

Gaming habits

54. Have you *in the last 12 months* bought lottery tickets or bet money on a game?
By game, we mean for example scratch cards, bingo, casino games, football pools, betting on horses or similar and games for money on the internet such as poker or online betting.

1 ☐ No → *Go on to question 57*
2 ☐ Yes

55. How much money have you used on gaming *in the last 7 days*?

Have played for ☐☐☐☐☐☐ **kronor**

1 ☐ Have not bet any money in the last 7 days

56. How many times in the last 12 months have you ...
Mark one alternative on each row.

	Never<br>1	1-2 times<br>2	3 times or more<br>3
a) ... tried to reduce your gaming?	☐	☐	☐
b) .. felt restless or irritated if you could not gamble?	☐	☐	☐
c) ... lied about how much you gamble?	☐	☐	☐

Alcohol habits

By alcohol we mean beer with medium or strong alcohol content, cider, wine, fortified wine and spirits.
Answer the questions as accurately and honestly as possible.

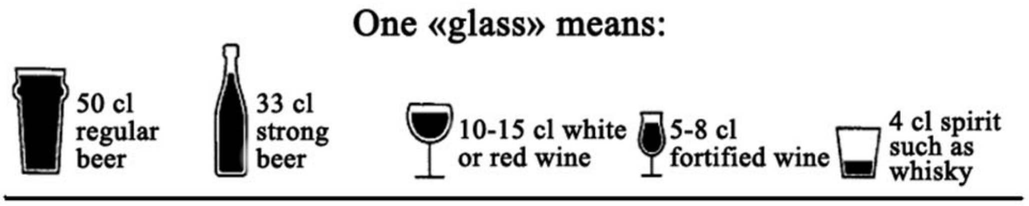

57. How often have you drunk alcohol in *the last 12 months*?

1 ☐ 4 times a week or more
2 ☐ 2-3 times a week
3 ☐ 2-4 times a month
4 ☐ Once a month or less
5 ☐ Never → *Go on to question 62*

58. How many "glasses" (see example) do you drink on a typical day when you drink alcohol?

1 ☐ 1-2
2 ☐ 3-4
3 ☐ 5-6
4 ☐ 7-9
5 ☐ 10 or more
6 ☐ Don't know

59. How often do you drink six "glasses" or more at a time?

1 ☐ Daily or almost every day
2 ☐ Every week
3 ☐ Every month
4 ☐ Less than once a month
5 ☐ Never

60. How often in *the last 12 months* have you drunk so much alcohol that you have become intoxicated?

1 ☐ Daily or almost every day
2 ☐ A few times a week
3 ☐ Once a week
4 ☐ 2-3 times a month
5 ☐ Once a month
6 ☐ Once or a few times every six months
7 ☐ Less often or never

61. Would you like to reduce your alcohol consumption?

1 ☐ Yes, and I believe I will be able to do this myself
2 ☐ Yes, but I need support
3 ☐ No

Economic circumstances

62. If you should suddenly find yourself in an unforeseen situation where you had to find 15 000 kronor in *one week*, would you manage it?

1 ☐ Yes
2 ☐ No

63. During *the last 12 months*, have you ever had difficulty in managing the regular expenses for food, rent, bills etc.?

1 ☐ No
2 ☐ Yes, once
3 ☐ Yes, more than once

Work and employment

64. What is your present form of employment?
More than one answer can be given.

1 ☐ Work as an employee → ☐☐☐ **% of full-time**
1 ☐ Self-employed
1 ☐ Leave of absence or parental leave
1 ☐ Studying, training
1 ☐ Labour market measures
1 ☐ Unemployed
1 ☐ Retired
1 ☐ Sickness benefit (disability pension)
1 ☐ Long term sick leave (more than 3 months)
1 ☐ Taking care of own household
1 ☐ Other, write in the rectangle: ________

65. a) What is/was your main job?

If you are not at work at the moment, state what work you have mainly had.
Try to give as detailed a work title as possible.

For example: Instead of assistant, write purchasing assistant. Please use block capitals!

Example: Instead of driver put for example:

B U S S C H A U F F Ö R

Your job (*if possible, please, write your main job in Swedish)*:

☐☐☐☐☐☐☐☐☐☐☐☐☐☐☐☐☐☐☐☐

b) What are/were your main tasks?

Describe your main working tasks. For example, if you are a project manager or similar, write what you do - such as "responsible for improving the working environment in social care for the elderly". If you are a factory worker, describe what you do or make.

Below are some questions for those of you who are gainfully employed (you should also answer if you are on sick leave or leave of absence or parental leave). If you are not gainfully employed, go on to question 68.

66. How satisfied are you with your tasks at work?

1 ☐ Very satisfied
2 ☐ Quite satisfied
3 ☐ Neither satisfied nor unsatisfied
4 ☐ Quite unsatisfied
5 ☐ Very unsatisfied

67. Are you worried about losing your job in the coming year?

1 ☐ Yes
2 ☐ No

68. a) Is there anyone close to you who is old or sick and who you help with day to day tasks, look after or care for?

1 ☐ Yes
2 ☐ No → *Go on to question 69*

b) On average, how many hours a week does this mean for you?

☐☐ **hours per week**

Security and social relations

69. Do you ever avoid going out alone for fear of being assaulted, robbed or otherwise victimised?

1 ☐ No
2 ☐ Yes, sometimes
3 ☐ Yes, often

70. a) Have you, during *the last 12 months* been <u>subjected to physical violence</u>?

1 ☐ Yes
2 ☐ No → *Go on to question 71*

b) Where did the violence occur?
More than one answer can be given.

1 ☐ At work/in school
1 ☐ In the home
1 ☐ In someone else's home/residential area
1 ☐ In a public place/place of entertainment
1 ☐ On or in connection with a train, bus, metro
1 ☐ Somewhere else

71. Have you, during *the last 12 months* been subjected to a <u>threat or menace</u> of violence, so that you were scared?

1 ☐ Yes
2 ☐ No

72. During *the last three months,* have you been treated in a way that made you feel humiliated?

1 ☐ No *Go on to question 74*
2 ☐ Yes, sometimes
3 ☐ Yes, several times

73. Was the offensive or abusive behaviour/treatment connected with any of the following?
More than one answer can be given.

1 ☐ Ethnic origin
1 ☐ Gender
1 ☐ Sexual orientation
1 ☐ Age
1 ☐ Disability
1 ☐ Religion
1 ☐ Skin colour
1 ☐ Appearance
1 ☐ Gender identity and/or gender expression
1 ☐ Other
1 ☐ Don't know

74. Do you have anyone you can share your innermost feelings with and confide in?

1 ☐ Yes
2 ☐ No

75. Can you get help from any person or persons if you have practical problems *or* are ill?
E.g. get advice, borrow things, help with shopping, repairs etc.

1 ☐ Yes, always
2 ☐ Yes, most of the time
3 ☐ No, mostly not
4 ☐ No, never

76. Do you think that people generally can rely on other people?

1 ☐ Yes
2 ☐ No

77. Have you taken part in any of the following activities *in the last 12 months*?
More than one answer can be given.

1 ☐ Study circle/course at your workplace
1 ☐ Study circle/course in your free time
1 ☐ Trade union meeting
1 ☐ Other association meeting
1 ☐ Theatre/cinema
1 ☐ Art exhibition
1 ☐ Religious gathering
1 ☐ Sporting event
1 ☐ Written to the editor at newspapers/periodicals
1 ☐ Demonstration of some kind
1 ☐ Public entertainment *e.g. night club, dance or similar*
1 ☐ Large family gathering
1 ☐ Private party at someone's home
1 ☐ None of the above

78. How much confidence do you have in the following institutions/politicians in society?
Mark one alternative on each row.

	Very much<br>1	Quite a lot<br>2	Not very much<br>3	None at all<br>4	Have no opinion<br>5
a) Health care	☐	☐	☐	☐	☐
b) Care for the elderly	☐	☐	☐	☐	☐
c) Child care	☐	☐	☐	☐	☐
d) Schools	☐	☐	☐	☐	☐
e) The police	☐	☐	☐	☐	☐
f) Social services	☐	☐	☐	☐	☐
g) Employment offices	☐	☐	☐	☐	☐
h) The social insurance agency Försäkringskassan	☐	☐	☐	☐	☐
i) Riksdagen (parliament)	☐	☐	☐	☐	☐
j) Politicians in your county council/region	☐	☐	☐	☐	☐
k) Politicians in your municipality	☐	☐	☐	☐	☐
l) Trade unions	☐	☐	☐	☐	☐

Background

79. What year were you born?

Year: 1 9 ☐ ☐

80. Are you male or female?

1 ☐ Male
2 ☐ Female

81. What is your sexual orientation?

1 ☐ Heterosexual
2 ☐ Bisexual
3 ☐ Homosexual
4 ☐ Uncertain of my sexual orientation

82. How do you live?

1 ☐ Own detached/terraced house
2 ☐ Own apartment
3 ☐ Rented apartment
4 ☐ Lodger, student apartment/room
5 ☐ Other

83. a) Who do you share a home with?
i.e. who do you live with for most of the week.
You can mark more than one alternative.

1 ☐ Nobody
1 ☐ Parents/siblings
1 ☐ Spouse or partner
1 ☐ Other adult
1 ☐ Children

b) How old are the children who live with you?

1 ☐ 0-6 years
1 ☐ 7-12 years
1 ☐ 13-17 years
1 ☐ 18 or older

c) Do any of these children have one or more functional disabilities?
Reduced functional ability means, for example, restricted movement, dyslexia, reduced eyesight or hearing. It might also mean ADHD, epilepsy or diabetes.

1 ☐ Yes
2 ☐ No

d) How old is the child/children with one or more functional disabilities?

You can mark several alternatives

1 ☐ 0-6 years
1 ☐ 7-12 years
1 ☐ 13-17 years
1 ☐ 18 or older

Functional ability, health problems and mental health

84. By placing a tick in one box in each group below, please indicate which statements best describe your own health state today.

A Mobility

1 ☐ I have no problems in walking about
2 ☐ I have some problems in walking about
3 ☐ I am confined to bed

B Self-Care

1 ☐ I have no problems with self-care
2 ☐ I have some problems washing or dressing myself
3 ☐ I am unable to wash or dress myself

C Usual Activities (e.g. work, study, housework, family and leisure activities)

1 ☐ I have no problems with performing my usual activities
2 ☐ I have some problems with performing my usual activities
3 ☐ I am unable to perform my usual activities

D Pain/Discomfort

1 ☐ I have no pain or discomfort
2 ☐ I have moderate pain or discomfort
3 ☐ I have extreme pain or discomfort

E Anxiety/Depression

1 ☐ I am not anxious or depressed
2 ☐ I am moderately anxious or depressed
3 ☐ I am extremely anxious or depressed

Domestic work

85. How many hours a week on <u>average</u> do you spend working at home that was not paid work?
E.g. taking care of children, nursing relatives, buying the groceries, cooking, paying the bills, washing the laundry, cleaning, taking care of a car, house or garden.

1 ☐ 0 – 2 hours a week
2 ☐ 3 – 10 hours a week
3 ☐ 11 – 20 hours a week
4 ☐ 21 – 30 hours a week
5 ☐ 31 hours a week or more

86. Do you find the domestic work burdensome?

1 ☐ Never
2 ☐ Seldom
3 ☐ Sometimes
4 ☐ Mostly
5 ☐ Always

Lifestyle habits

87. How often do you eat breakfast, lunch, dinner and supper?
Mark one alternative on each row.

	Daily or mostly daily<br>1	A few times a week<br>2	Seldom or never<br>3
Breakfast	☐	☐	☐
Lunch	☐	☐	☐
Dinner	☐	☐	☐
Supper	☐	☐	☐

If you have visited a health care centre or similar in the last 3 months answer questions 88 and 89, otherwise go on to question 90.

88. At your most recent visit to a health care centre or similar, did the personnel ask about your:
Mark one alternative on each row.

	No<br>1	Yes<br>2
Food habits	☐	☐
Exercise habits	☐	☐
Smoking habits	☐	☐
Snuff habits	☐	☐
Alcohol habits	☐	☐

89. At your most recent visit to a health care centre or similar, did you receive any advice to change your:
Mark one alternative on each row.

	No<br>1	Yes<br>2
Food habits	☐	☐
Exercise habits	☐	☐
Smoking habits	☐	☐
Snuff habits	☐	☐
Alcohol habits	☐	☐

If you have visited or been admitted to hospital in the last 3 months answer questions 90 and 91, otherwise go on to question 92.

90. At your most recent visit to hospital, did the personnel ask about your:
Mark one alternative on each row.

	No<br>1	Yes<br>2
Food habits	☐	☐
Exercise habits	☐	☐
Smoking habits	☐	☐
Snuff habits	☐	☐
Alcohol habits	☐	☐

91. At your most recent visit to hospital, did you receive any advice to change your:
Mark one alternative on each row.

	No<br>1	Yes<br>2
Food habits	☐	☐
Exercise habits	☐	☐
Smoking habits	☐	☐
Snuff habits	☐	☐
Alcohol habits	☐	☐

Relations and conditions during childhood

92. The following statements express social cohesion or confidence in people in the area where we live. To what extent do the following statements apply to your area?

a) You can rely on the people who live in the area.

1 ☐ Applies very well
2 ☐ Applies quite well
3 ☐ Does not apply particularly well
4 ☐ Does not apply at all

b) You can feel safe in this area and secure that you will not be assaulted or subjected to threats.

1 ☐ Applies very well
2 ☐ Applies quite well
3 ☐ Does not apply particularly well
4 ☐ Does not apply at all

93. During the last 3 months, have you experienced that someone has made you feel proud?

1 ☐ No
2 ☐ Yes, once or twice
3 ☐ Yes, several times

94. Have you during the past three months felt that someone has treated you in a condescending manner?

1 ☐ No
2 ☐ Yes, once or twice
3 ☐ Yes, several times

95. Were you treated in a condescending manner while you were <u>growing up</u>, in school or at home for example?

1 ☐ No
2 ☐ Yes, sometimes
3 ☐ Yes, often

96. Did your family have any financial difficulties while you were <u>growing up</u>?

1 ☐ Yes, all the time I was growing up
2 ☐ Yes, most of the time I was growing up
3 ☐ Yes, some of the time I was growing up
4 ☐ No

Finally here are some questions for you as a gainfully employed (this also includes you who are on sick leave, leave of absence or parental leave)

97. Who is your <u>main</u> employer?
Mark at least one alternative.

1 ☐ Myself or a family member
2 ☐ Private employer
3 ☐ Municipality
4 ☐ County council
5 ☐ Central Government
6 ☐ Other

98. How many hours do you work on <u>average</u> in a normal working week?
State a whole number of hours.

☐☐ **Hours per week**

99. Would you like to work more or less per week?

1 ☐ I would like to work less
2 ☐ I would like to work more
3 ☐ Neither more nor less

100. How much physical movement or exertion do you get in your work?
Mark at least one alternative.

1 ☐ **Sedentary work:** You have a predominantly sedentary work and do not walk very much.
2 ☐ **Light but somewhat mobile work:** Your work means that you walk quite a lot but do not carry or lift heavy items.
3 ☐ **Moderately heavy work:** You walk a lot and also lift heavy items.
4 ☐ **Heavy work:** You have heavy physical work, lift heavy items and are involved in frequent physical exertion.

101. How often do the following events occur in your work?

a) I am exposed to noise (must raise my voice in conversation)

1 ☐ Every day
2 ☐ Some days a week
3 ☐ Less often
4 ☐ Never

b) I perform repetitive and one-sided working movements

1 ☐ Every day
2 ☐ Some days a week
3 ☐ Less often
4 ☐ Never

102. How many days sick leave have you had in the last 12 months?
Either with or without a doctor's certificate.

1 ☐ Have not had sick leave
2 ☐ 1–28 days
3 ☐ 29-59 days
4 ☐ 60-89 days
5 ☐ 90 days or more

103. If you have had continuous sick leave for 29 days or more in the last 12 months: Why were you on sick leave?
You can mark several alternatives.

1 ☐ Have not had continuous sick leave for 29 days or more
1 ☐ Back, neck, joint or muscle problems
1 ☐ Burnout, exhaustion depression, stress
1 ☐ Other mental problems (including sleep problems)
1 ☐ Cardiovascular disease, stroke
1 ☐ Accident, injury
1 ☐ Other

If there is anything else you would like to tell us, you are welcome to write it here. What you write will be sent to the Swedish National Institute of Public Health and your county council together with your answers.

THANK YOU FOR TAKING THE TIME TO FILL IN THIS QUESTIONNAIRE!

Place the questionnaire in the return envelope attached.
